# Profile of the HIV Epidemic in Cape Verde: Molecular Epidemiology and Drug Resistance Mutations among HIV-1 and HIV-2 Infected Patients from Distinct Islands of the Archipelago

**DOI:** 10.1371/journal.pone.0096201

**Published:** 2014-04-24

**Authors:** Isabel Inês M. de Pina-Araujo, Monick L. Guimarães, Gonzalo Bello, Ana Carolina P. Vicente, Mariza G. Morgado

**Affiliations:** 1 Laboratório de AIDS & Imunologia Molecular, Instituto Oswaldo Cruz, Fundação Oswaldo Cruz, Rio de Janeiro, RJ, Brasil; 2 Departamento de Ciência e Tecnologia, Universidade de Cabo Verde, Praia, Santiago, Cabo Verde; 3 Laboratório de Genética Molecular de Microorganismos, Instituto Oswaldo Cruz, Fundação Oswaldo Cruz, Rio de Janeiro, RJ, Brasil; Centro de Biología Molecular Severo Ochoa (CSIC-UAM), Spain

## Abstract

HIV-1 and HIV-2 have been detected in Cape Verde since 1987, but little is known regarding the genetic diversity of these viruses in this archipelago, located near the West African coast. In this study, we characterized the molecular epidemiology of HIV-1 and HIV-2 and described the occurrence of drug resistance mutations (DRM) among antiretroviral therapy naïve (ARTn) patients and patients under treatment (ARTexp) from different Cape Verde islands. Blood samples, socio-demographic and clinical-laboratory data were obtained from 221 HIV-positive individuals during 2010–2011. Phylogenetic and bootscan analyses of the *pol* region (1300 bp) were performed for viral subtyping. HIV-1 and HIV-2 DRM were evaluated for ARTn and ARTexp patients using the Stanford HIV Database and HIV-GRADE e.V. Algorithm Homepage, respectively. Among the 221 patients (169 [76.5%] HIV-1, 43 [19.5%] HIV-2 and 9 [4.1%] HIV-1/HIV-2 co-infections), 67% were female. The median ages were 34 (IQR = 1–75) and 47 (IQR = 12–84) for HIV-1 and HIV-2, respectively. HIV-1 infections were due to subtypes G (36.6%), CRF02_AG (30.6%), F1 (9.7%), URFs (10.4%), B (5.2%), CRF05_DF (3.0%), C (2.2%), CRF06_cpx (0.7%), CRF25_cpx (0.7%) and CRF49_cpx (0.7%), whereas all HIV-2 infections belonged to group A. Transmitted DRM (TDRM) was observed in 3.4% (2/58) of ARTn HIV-1-infected patients (1.7% NRTI, 1.7% NNRTI), but not among those with HIV-2. Among ARTexp patients, DRM was observed in 47.8% (33/69) of HIV-1 (37.7% NRTI, 37.7% NNRTI, 7.4% PI, 33.3% for two classes) and 17.6% (3/17) of HIV-2-infections (17.6% NRTI, 11.8% PI, 11.8% both). This study indicates that Cape Verde has a complex and unique HIV-1 molecular epidemiological scenario dominated by HIV-1 subtypes G, CRF02_AG and F1 and HIV-2 subtype A. The occurrence of TDRM and the relatively high level of DRM among treated patients are of concern. Continuous monitoring of patients on ART, including genotyping, are public policies to be implemented.

## Introduction

Human Immunodeficiency Virus (HIV), the etiologic agent of Acquired Immunodeficiency Syndrome (AIDS), remains a challenge in the 21st century, even more than 30 years after its discovery. HIV is a highly divergent virus with two types described so far, HIV-1 and HIV-2 [1], [2]. HIV-1 includes four groups, M, O, N and P [3]; the M group is responsible for the majority of infections worldwide and is divided into nine subtypes (A–D, F–H, J and K), six sub-subtypes (A1–A4, F1–F2), and a large number of unique (URF) and circulating (CRF) recombinant forms [4]. Conversely, HIV-2 is more restricted to West Africa and can be classified into 8 groups (A–H), with only one CRF01_AB [5] and a novel HIV_2 variant, recently identified in the Ivory Coast [6].

At the end of 2012, 35.3 million people were living with HIV, and approximately 10.6 million people received antiretroviral treatment (ART) [7]. The number of Africans under ART increased from one to seven million between 2005 and 2012, whereas the number of AIDS-related deaths was reduced by 32% in the same period [8]. Studies demonstrated the efficacy of ART not only as a treatment but also in prevention [9]. The increase of ART availability also brings a challenge in monitoring the emergence of drug resistance mutations (DRM) on naïve and treated patients, particularly in countries with limited capacities to implement genotyping tests. The fully described differences on the susceptibility of HIV-1 and HIV-2 to the currently available classes of antiretroviral drugs are challenges for patient management in countries where the two viruses co-circulate [10].

Cape Verde is an archipelago located in the Atlantic Ocean, 445 km from the coast of West Africa, and was discovered by Portuguese explorers in 1640. The independence of the archipelago was established on July 5, 1975. In 2000, the population was estimated at 491,875 inhabitants with a mean age of 26.8 years [11]. In West Africa, where Cape Verde is located, two types of HIV (HIV-1 and HIV-2) co-circulate, and within HIV-1, CRF02_AG (50%) predominates, followed by subtype G (28%), URFs (8%) and subtype A (4%) [12]. However, for many countries in this region, including Cape Verde, little to no information is available for HIV-1 and HIV-2 molecular diversity. Serological tests for HIV-1 and HIV-2 antibodies started in Cape Verde in 1987, and both viruses have been detected since then. Moreover, the first isolation of HIV-2 was from an AIDS patient from Cape Verde [11]. Epidemiological data from Cape Verde [11], [13] indicates that since the first detections of HIV in Cape Verde, the accumulated number of seropositive patients increased to 3,816 by 2011, within which 1,443 developed AIDS and 847 died from AIDS-related diseases. Based on the Demographic and Reproductive Health Survey [14] conducted in Cape Verde in 2005, the overall HIV prevalence in Cape Verde was estimated at 0.8%, being higher among men (1.1%) than among women (0.4%) [13]. The highest prevalence (1.7%) was observed in Praia, the capital of the country. Free ART for AIDS patients, including pregnant women to prevent mother-to-child transmission, started to be available in Cape Verde only at the end of 2004 [15]. The protocol of HIV-1 regimen follows the WHO recommendation, including nucleoside/nucleotide reverse transcriptase inhibitors (NRTI), non-nucleoside reverse transcriptase inhibitors (NNRTI) and protease inhibitors (PI) as follows: 2NRTI+1NNRTI for first line and 2NRTI+1PI for second line [15]. For HIV-2 infected patients the protocol used in Cape Verde includes 2NRTI+1PI [16].

Because of its strategic location, Cape Verde is a hub of communication between Africa, Europe and the Americas. Business, tourism and political discussions facilitated by transportation to and from Cape Verde put this country in an important role in the infectious disease movement, including HIV/AIDS, as previously documented for the global dispersion of the HIV-2 A [17]. Although one molecular epidemiological study regarding HIV diversity and drug resistance in Cape Verde is available so far, it was based on analyses of a low number of HIV-1 (n = 27) and HIV-2 (n = 14) infected patients residing on a single island (Santiago) [18]. According to this study, subtype G was the most prevalent HIV-1 clade (48%) and 12% of transmitted drug resistance mutations were detected among the HIV-1 untreated patients.

Thus, the objective of this study was to characterize the molecular epidemiological profile of HIV-1 and HIV-2 circulating in the Cape Verde archipelago, its association with local socio-demographic and clinical data and to describe the occurrence of DRM among ARTn and ARTexp patients from seven Cape Verde islands. Because of the strategic position of Cape Verde, our results can contribute to the understanding of HIV dynamics both locally and globally in addition to defining public health polices in HIV/AIDS.

## Materials and Methods

### Ethics

The study was approved by the Cape Verde National Ethics Committee in health research and all participants in the study signed informed consent. Written consents of relatives or guardians were obtained on behalf of the children and adolescents included in the study. This study was conducted under agreement with the University of Cape Verde, the Ministry of Health in Cape Verde and the Oswaldo Cruz Foundation in Brazil.

### Study Population and Sample Processing

As part of a large national cross-sectional study to assess the molecular epidemiology of HIV in Cape Verde, blood samples, socio-demographic and clinical-laboratory data were obtained from 221 HIV positive patients during 2010 and 2011 in seven of the nine Cape Verde inhabited islands. This convenience sample included HIV positive outpatients under clinical and laboratory monitoring in distinct Cape Verde public health services, independent of the age, time of infection, treatment and comorbidities. Hospitalized patients were not eligible for the study. CD4 counts were determined by Facscount software (BD FACSCount Instrument). Blood samples were collected in EDTA tubes, locally processed, and transported to the Laboratory of ELISA in the Dr. Agostinho Neto Central Hospital, Praia, for storage at −20°C until transportation in dry ice to the Laboratory of AIDS and Molecular Immunology at the Oswaldo Cruz Institute (IOC/FIOCRUZ), Brazil, according to international biosafety rules, for molecular analyses.

### HIV Subtyping

RNA and DNA were extracted, respectively, from 99 HIV-1 plasma and 70 HIV-1, 43 HIV-2 and 9 HIV-1/HIV-2 blood samples, using commercial kits (QIAmp Viral RNA and QIAmp DNA Blood, QIAGEN, Valencia, CA, respectively), following manufacturer’s instructions. In general, the success of amplification and sequencing was about 80% for HIV-1 and HIV-2 samples. cDNA from HIV-1 samples were obtained by RT-PCR using an in-house method followed by a nested PCR protocol covering a fragment of approximately 1300 bp of the *pol* region including the Protease (PR; aa 1–99) and part of the Reverse Transcriptase (RT; aa 1–540). Because of the complexity of the studied samples, different primer sets covering this region were used. Outer and inner primer sets and PCR conditions are available by request. HIV-2 proviral DNA amplification was performed as described elsewhere [19]. Additional HIV-2 primers (H2CVp1R [3408–3427]: TATATRTATCTTTTTCCTGG and H2CVp2F [3095–3113]: ATCTGTGAAAAAATGGAAA) were defined and used for sequencing. RT-PCR and PCR products were purified using the Illustra GFX PCR DNA Kit (GE Healthcare, Inc., Little Chalfont, Buckinghamshire, UK) and sequenced using the v.3.1 Cycle Sequencing Ready Reaction Kit (Applied Biosystems, Carlsbad, CA) with an automated ABI 3100 Genetic Analyzer (Applied Biosystems, Carlsbad, CA). Sequence electropherograms were visualized and assembled using the Seqman program (DNASTAR; Lasergene, Madison, Wis., USA). Sequences were aligned by the ClustalW algorithm implemented in the Mega v5.0 package [20] and a final alignment of 966 nucleotides (nucleotides 2256–3222 relative to HXB2) was obtained. Subtype determination was performed by: 1) the REGA program [21], [22]; 2) Neighbor-Joining (NJ) phylogenetic analyses using MEGA program [20], and 3) Bootscan analyses with Simplot 3.5.1 software [23]. NJ phylogenetic trees were inferred under the Kimura 2-parameter (K2-P) nucleotide substitution model and reliability of the obtained tree topology was estimated with the bootstrap method based on 1000 re-samplings. In Bootscan analyses, bootstrap values supporting branching with reference sequences of all HIV-1 group M subtypes were determined in NJ trees constructed using the K2-P model, based on 100 re-samplings, with a sliding window of 200 bp moving in steps of 20 bp. Reference strains of HIV-1 group M subtypes and CRFs and HIV-2 groups A and B were retrieved from the Los Alamos HIV database [4]. The sequences were submitted to Genbank and the accession numbers are KJ395593–KJ395726 for HIV-1 and KJ395727–KJ395756 for HIV-2 sequences.

### Drug-Resistance Mutation Analyses

Sequences from HIV-1 and HIV-2 were evaluated for DRM for ARTn and ARTexp. For HIV-1 sequences, the analysis was performed through the Stanford HIV Database for Transmitted DRM (TDRM/CPR Tool) Code Version 6.0 and DRM (HIVdb Program) Version 6.3.1 for naïve and treated patients, respectively [24], [25]. For HIV-2 ART-naive and ART-patients, analyses was performed according to the HIV-GRADE e.V. Algorithm Homepage for HIV-2 sequences [26].

### Statistical Analyses

The clinical, laboratory and epidemiological data were analyzed as categorical or continuous variables using contingency tables (Chi-squared and Fisher Exact Test) and non-parametrical methods, respectively, through the GraphPad Prism v.6.01 software.

To evaluate the differential characteristics of the HIV epidemic in Cape Verde, the country was divided in two major areas (Barlavento and Sotavento) that were further divided according to the most representative islands, such as São Vicente and other Barlavento islands and Santiago and other Sotavento islands, respectively. The data from Santiago island was further divided in the capital of the country (Praia), which concentrates 26.8% of the total population from Cape Verde and Inner Santiago (Santiago island without Praia).

## Results

From 2010 to 2011, a total of 221 HIV positive individuals from seven Cape Verde islands were included in the present study. Among them, 169 (76.5%) were infected with HIV-1, 43 (19.5%) with HIV-2 and 9 (4.1%) with both types of HIV. These serological data coincide with the percentages of infections because of the HIV types circulating at present in Cape Verde [13]. The major socio-demographic and clinical data distributed according to the type of virus (HIV-1, HIV-2 and HIV-1+2 co-infection) are listed in Table 1. More than 65% of the individuals were female without differences among the HIV-1, HIV-2 and HIV-1+2 groups. A higher frequency of older individuals (>49 years) was observed in the HIV-2 infected group (44.2%) compared with the HIV-1 group (16.6%) (p = 0.0005). A higher median age was also detected in the HIV-2 group [47 (IQR = 12–84)] compared with the HIV-1 group [34 (IQR = 1–75)] [p<0.0001]. The frequency of infected children due to vertical transmission was higher for HIV-1 compared to HIV-2 (p = 0.03). The two viruses were present in patients from all Cape Verde islands in the same proportions, with the exception of Santiago’s island. The frequency of HIV-2 infections is significantly higher in Praia (71.4%) than in Inner Santiago (28.6%) (p = 0.03). Half of the HIV-1 and HIV-2 infected patients were treated for a median time of 26.0 months (IQR = 1–93) for HIV-1 and 35.5 months (IQR = 17–68) for HIV-2. More than 55% of the HIV-1- and HIV-2-infected individuals displayed CD4 T cell counts above 350 cells/mm^3^.

**Table 1:** Socio-demographic and clinical data of 221 HIV seropositive individuals from Cape Verde distributed according to the type of virus.

Variables	HIV-1	HIV-2	HIV-1+2	P value* (95%)
N (%)	169 (76. 5)	43 (19. 5)	9 (4.1)	
**Gender N (%)**				1.0
Female	112 (66.3)	29 (67.4)	7 (77.8)	
Male	54 (32.0)	14 (32.6)	2 (22.2)	
Unknown	3 (1.8)			
**Age Group (years) N (%)**				0.005
0–15	28 (16.6)	2 (4.7)		
16–29	29 (16.6)	3 (7.0)	1 (11.1)	
30–49	85 (50.3)	19 (44.2)	7 (77.8)	
>49	28 (16.6)	19 (44.2)	1 (11.1)	
**Year of Serodiagnostic N (%)**				0.739
Before 2003	7 (4.1)	2 (4.7)	1 (11.1)	
2004–2007	50 (29.6)	8 (18.6)	1 (11.1)	
2008–2011	70 (41. 4)	15 (34.9)	4 (44.4)	
Unknown	42 (24.9)	18 (41.9)	3 (33.3)	
**Residence (Region) N (%)**				0.85
** Barlavento**	**48 (28.4)**	**13 (30.3)**	**4 (44.4)**	
** Sotavento**	**121 (71.6)**	**30 (69.7)**	**5 (55.6)**	
***Barlavento (N = 65)***	***48***	***13***	***4***	***1.0***
S. Vicente	25 (52.1)	7 (53.8)	1 (25.0)	
Barlavento (without S. Vicente)	23 (47.9)	6 (46.2)	3 (75.0)	
***Sotavento (N = 156)***	***121***	***30***	***5***	***1.0***
Santiago	111 (91.7)	28 (93.3)	5 (100.0)	
Sotavento (Without Santiago)	10 (8.3)	2 (6.7)	-	
***Santiago (N = 144)***	***111***	***28***	***5***	***0.03***
Praia	53 (47.7)	20 (71.4)	2 (40.0)	
Inner Santiago	58 (52.3)	8 (28.6)	3 (60.0)	
**Transmission N (%)**				0.03
Heterosexual	101 (59.8)	26 (60.5)	6 (66.7)	
Vertical	27 (16.0)	1 (2.3)	-	
Others	2 (1.2)	1 (2.3)	1 (11.1)	
Unknown	39 (23.1)	15 (34.9)	2 (22.2)	
**ARV N (%)**				1.0
Yes	92 (54.4)	21 (48.8)	4 (44.4)	
No	73 (43.2)	16 (37.2)	3 (33.3)	
Unknown	4 (2.4)	6 (14.0)	2 (22.2)	
**CD_4_ T cell count (cells/mm^3^) N (%)**				0.5
<350	47 (27.8)	16 (37.2)	5 (55.6)	
350–500	37 (21.9)	7 (16.3)	1 (11.1)	
>500	62 (36.7)	17 (39.5)	1 (11.1)	
Unknown	23 (13.6)	3 (7.0)	2 (22.2)	

*Only for HIV-1 and HIV-2 and without unknown value.

### HIV-1 and HIV-2 Genetic Characterization

**Figure 1:**
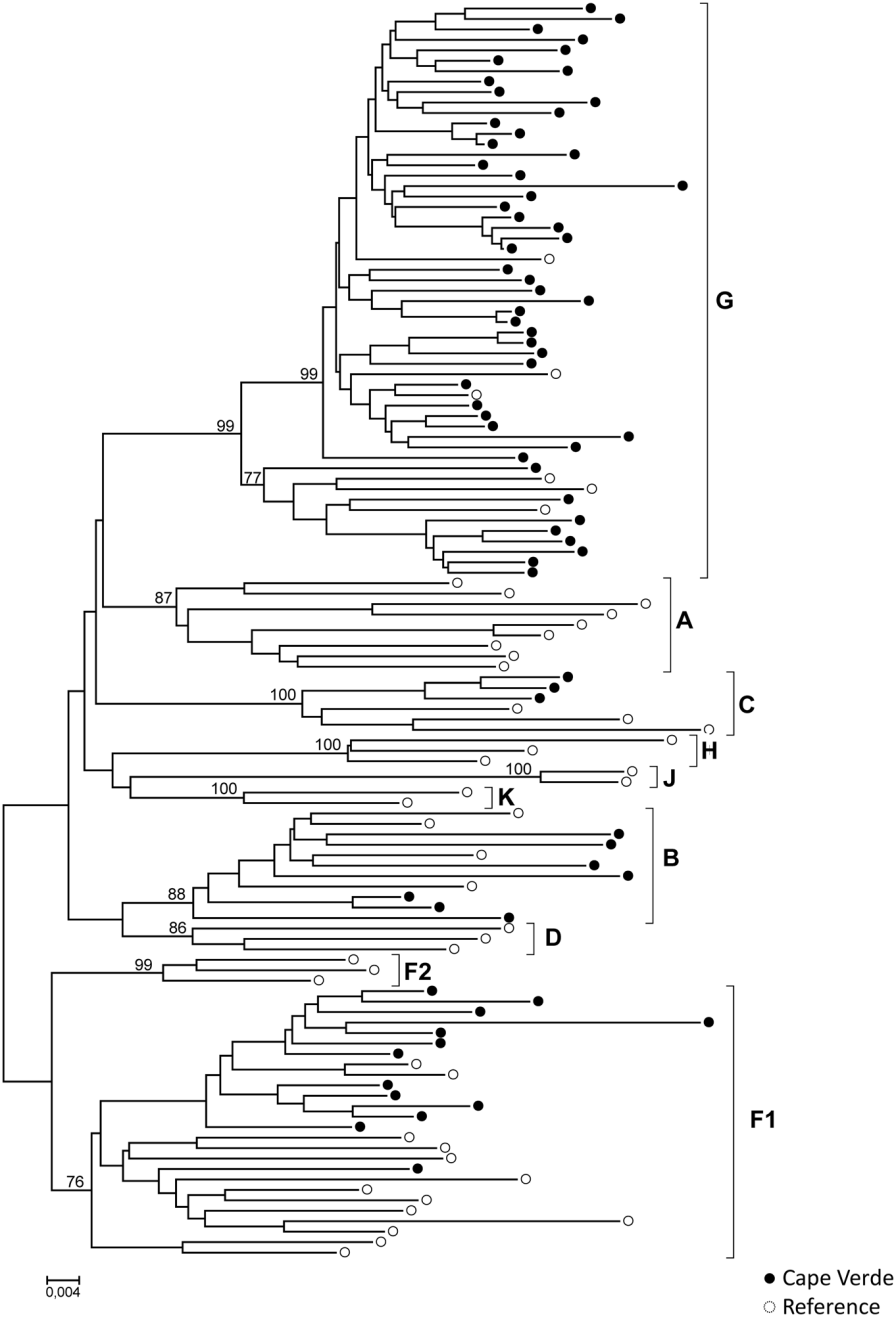
Phylogenetic tree analysis of the *pol* region of HIV-1 “pure subtype” samples from Cape Verde. The phylogenetic inferences were performed by the Neighbor-Joining algorithm under the Kimura-2 parameter nucleotide substitution model using the MEGA v5.0 package. The scale represents the number of substitutions per site. Cape Verde and reference sequences are represented, respectively as black and white circles.

**Figure 2:**
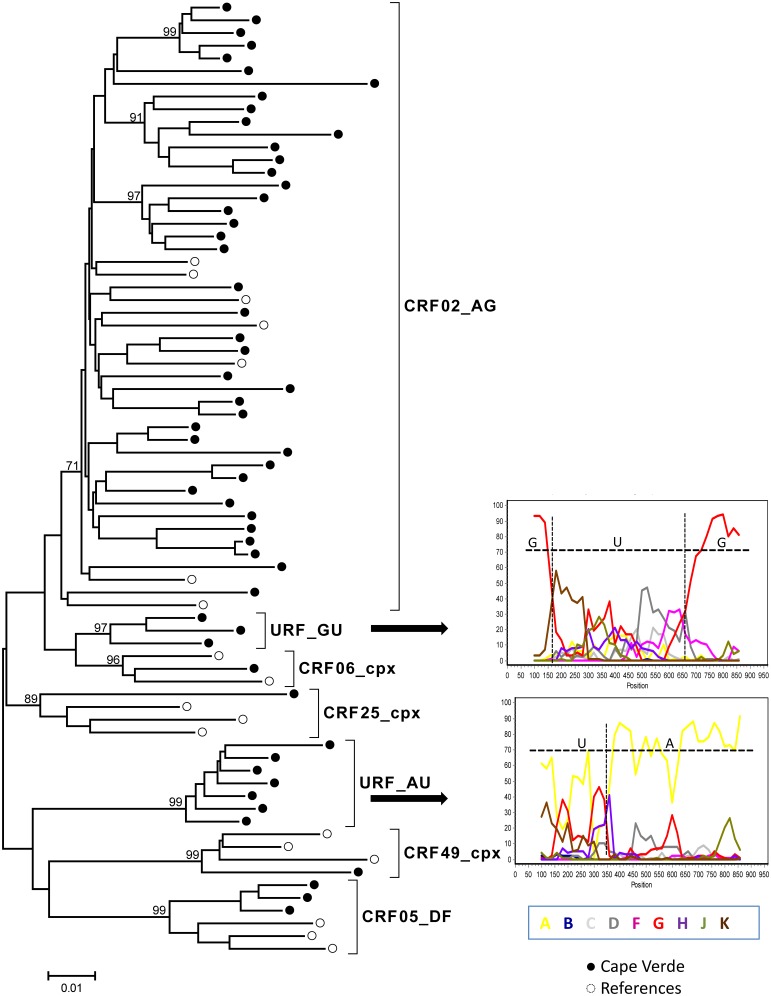
Phylogenetic tree analysis of the *pol* region of HIV-1 CRF and major URF samples from Cape Verde. The phylogenetic inferences were performed by the Neighbor-Joining algorithm under the Kimura-2 parameter nucleotide substitution model using the MEGA v5.0 package. The scale represents the number of substitutions per site. Cape Verde and reference sequences are represented, respectively as black and white circles. Bootscan analyses of HIV-1 major URF samples are displayed. Recombinant profiles were inferred using a sliding window of 200 bp, steps of 20 bp and the Kimura-2 parameters model using SimPlot 3.5.1 software. Reference samples corresponding to the major HIV-1 subtypes are indicated by different colors.

**Figure 3:**
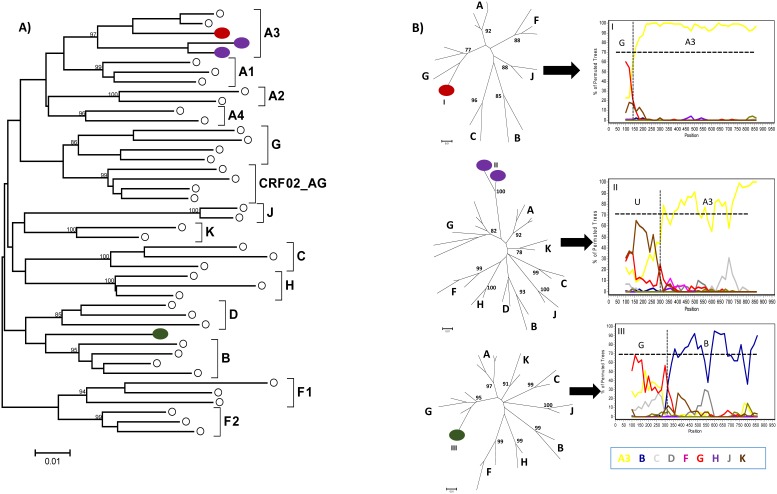
Phylogenetic tree and bootscan analyses of the *pol* region of HIV-1 URF samples from Cape Verde. (A) The phylogenetic inferences were performed by the Neighbor-Joining algorithm under the Kimura-2 parameter nucleotide substitution model using the MEGA v5.0 package. The scale represents the number of substitutions per site. Cape Verde and reference sequences are represented respectively as colored and white circles. (B) Recombinant profiles were inferred using a sliding window of 200 bp, steps of 20 bp and the Kimura-2 parameters model using SimPlot 3.5.1 software. Reference samples corresponding to the major HIV-1 subtypes are indicated by different colors. Partial NJ phylogenetic trees using K-2p model were performed for bootscan fragments under 70% and were represented as I - 150 bp and II and III - 300 bp.

**Figure 4:**
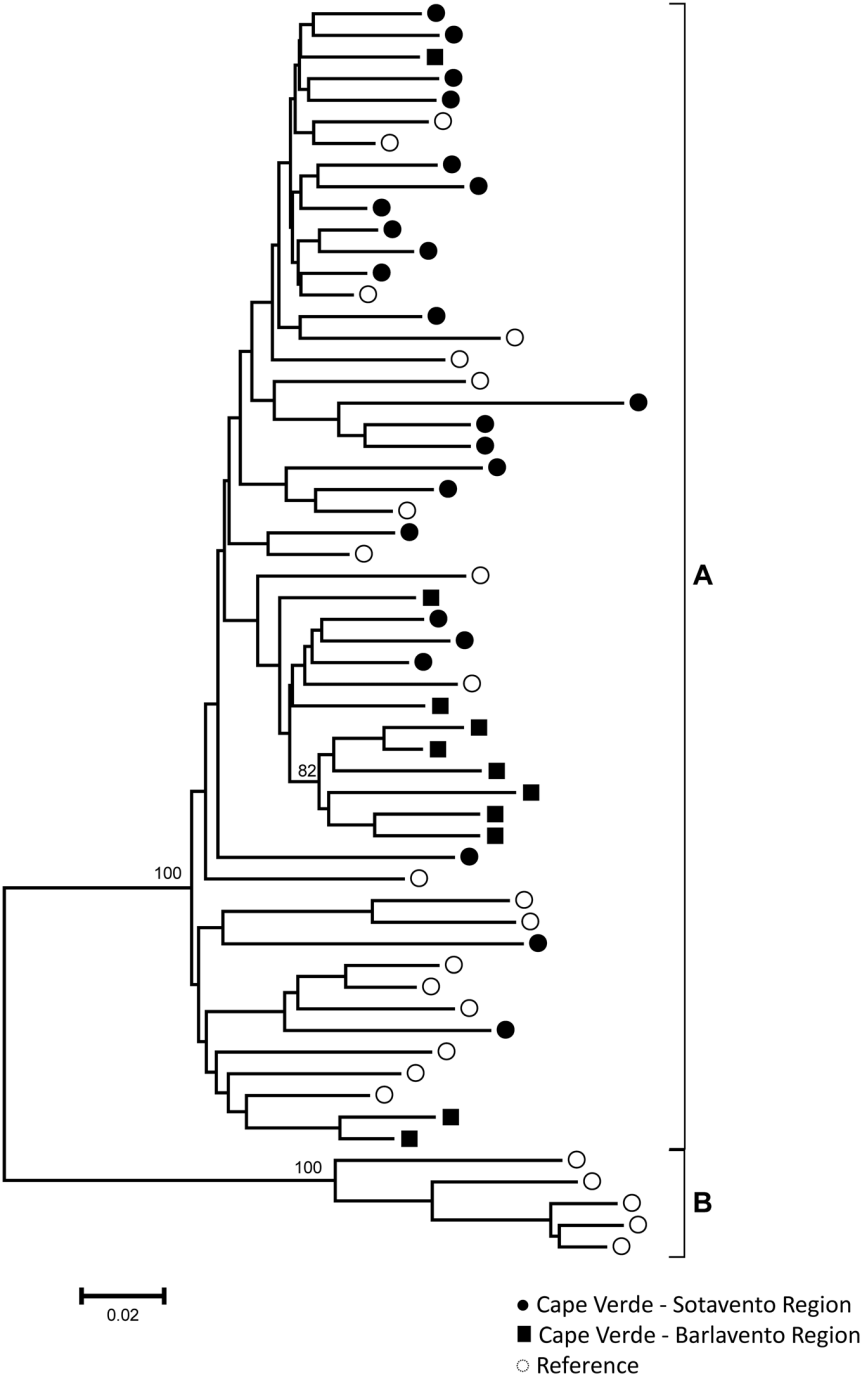
Phylogenetic tree analysis of the Cape Verde HIV-2 *pol* region samples (black circles and squares). The phylogenetic inferences were performed by the Neighbor-Joining algorithm under the Kimura-2 parameter nucleotide substitution model using the MEGA v5.0 package. Reference sequences from HIV-2 Group A and B are included and represented as white circles. The scale represents the number of substitutions per site.

*Pol* sequences were available for 134 HIV-1 and 34 HIV-2 specimens. For HIV-1 subtyping, the sequences were first submitted to REGA and later confirmed by phylogenetic and bootscan analyses. Several HIV-1 subtypes, URFs and CRFs-like samples were present in the Cape Verde study population. Phylogenetic tree comprising “pure” HIV-1 subtypes is presented in the Fig. 1. Figures 2 and 3 present phylogenetic analyses and recombinant profiles of the CRF and URF samples described in the present study. Overall, HIV-1 subtype G (36.6%) and CRF02_AG (30.6%) accounted for almost 70% of the cases, followed by subtype F1 (9.7%), URFs (10.4%), B (5.2%), CRF05_DF (3.0%), C (2.2%), CRF06_cpx (0.7%), CRF25_cpx (0.7%) and CRF49_cpx (0.7%). Notably, we observed the presence of two highly significantly supported URF clusters, confirmed by Simplot analyses, one with an AU mosaic structure comprising seven samples (5.2%) and another with a GU recombinant structure comprising three (2.2%) samples (Fig. 2). The A fragment of these seven URF_AU samples did not cluster with any of the previously described A sub-subtypes (A1-A4). Moreover, figure 3A shows tree additional A related genomes that clustered with significant bootstrap with the sub-subtype A3, and one sample mapping close to the B subtype branch but without bootstrap significance. Based on further Simplot analyses and partial phylogenetic trees of the Simplot fragments (Fig. 3B I-III) two recombinant genomes A3G (I) and A3U (II) were revealed as well as one BG (III). Overall, the intersubtype recombinant viruses comprise 46.1% of the HIV-1 samples analyzed in Cape Verde. All HIV-2 sequences belonged to group A. Most of these samples presented intermixed phylogenetic clustering independent of the geographic region; however, one highly supported cluster was composed by six of the eleven HIV-2 sequences obtained from HIV patients in Barlavento (Fig. 4).

### Socio-demographic and Clinical Data of HIV-1 Patients from Cape Verde According to Viral Subtype

To understand the role of viral variability in the local HIV-1 epidemic, the socio-demographic and clinical data were analyzed according to the major genetic variants circulating in Cape Verde (subtype G, CRF02_AG, subtype F1 and other viral forms) (Table 2). No differences could be detected for gender, age, year of diagnosis, route of transmission, use of ART or CD4 T cell counts among the patients infected with different variants. Some differences were observed, however, when individuals were divided according to their residence. Significant differences in the distribution of HIV-1 genetic variants were observed between Barlavento and Sotavento areas (p<0.0001), Sao Vicente and other Barlavento’s islands (p = 0.001), Santiago and other Sotavento’s islands (p = 0.003), and even Praia and Inner Santiago (p = 0.018). Such differences were predominantly driven by the high prevalence of subtype F1 in São Vicente (61%); subtype G in Inner Santiago (51%) and others islands from Sotavento (67%); and CRF02_AG in Praia (43%) and others islands from Barlavento (55%). Notably, six of seven subtype B samples identified in the present study were from patients recruited in Inner Santiago.

**Table 2:** Socio-demographic and clinical data of HIV-1 seropositive individuals from Cape Verde distributed according to the main viral genetic characterization in the *pol* region.

Variables	G	CRF02_AG	F	Others Forms	P value* (95%)
N (%)	49 (36.6)	41 (30.6)	13 (9.7)	31 (23.1)	
**Gender N (%)**					0.48
Female	36 (73.5)	26 (63.4)	7 (53.8)	21 (67.7)	
Male	12 (24.5)	14 (34.1)	6 (46.2)	10 (32.3)	
Unknown	1 (2.0)	1 (2.4)			
**Age Group (years) N (%)**					0.14
0–15	9 (18.4)	10 (24.4)		4 (12.9)	
16–29	10 (20. 4)	7 (17.1)	3 (23.1)	2 (6.5)	
30–49	24 (49.0)	20 (48. 8)	10 (76.9)	17 (54,8)	
>49	6 (12.2)	4 (9.8)	-	8 (25.8)	
**Year of Serodiagnostic N (%)**					0.84
Before 2004	2 (4.1)	1 (2.4)		3 (9.7)	
2004–2007	16 (32.7)	10 (24.4)	5 (38.5)	8 (25.8)	
2008–2011	20 (40.8)	19 (46.3)	6 (46.2)	14 (45.2)	
Unknown	22 (44.9)	11 (26.8)	2 (15.4)	6 (19.4)	
**Residence (Region) N (%)**					**<0.0001**
** Barlavento**	**6 (12.2)**	**14 (34.1)**	**12 (92.3)**	**6 (19.4)**	
** Sotavento**	**43 (87.8)**	**27 (65.9)**	**1 (7.7)**	**25 (80.6)**	
***Barlavento***					***0.001***
S. Vicente	1 (2.0)	3 (7.3)	11 (84.6)	3 (9.7)	
Others Islands	5 (10.2)	11 (26.8)	1 (7.7)	3 (9.7)	
***Sotavento***					***0.003***
Santiago	37 (75.5)	25 (61.0)		25 (61.0)	
Others Islands	6 (12.2)	2 (4.9)	1 (7.7)		
***Santiago***					**0.018**
Praia	14 (28.6)	18 (43.9)		10 (32.3)	
Inner Santiago	23 (46.9)	7 (17.1)		15 (48.4)	
**ARV n (%)**					0.41
Yes	24 (49.0)	22 (53.7)	5 (38.5)	19 (59.4)	
No	25 (51.0)	17 (41.5)	8 (61.5)	11 (35.5)	
Unknown		2 (4.9)		1 (3.2)	
**Transmission N (%)**					0.079
Heterosexual	29 (59.2)	17 (41. 5)	9 (69.2)	23 (74.2)	
Vertical	9 (18.4)	10 (24.4)		4 (12.9)	
Others		2 (4.9)	2 (15.4)		
Unknown	11 (22.4)	14 (34.1)	2 (15.4)	4 (12.9)	
**CD_4_ T cell count (cells/mm^3^) N (%)**					0.91
<350	19 (38.8)	11 (26.8)	3 (23.1)	11 (35.5)	
350–500	8 (16.3)	8 (19.5)	4 (30.8)	6 (19.4)	
>500	17 (34.7)	12 (29.3)	5 (38.5)	11 (35.5)	
Unknown	5 (10.2)	10 (24.4)	1 (7.7)	3 (9.7)	

*Without unknown value.

### Drug Resistance Mutations Among ARTn and ARTexp HIV-1 and HIV-2 Infected Individuals

Based on the polymerase sequences obtained from the HIV-1 and HIV-2 positive individuals from Cape Verde, we also evaluated the occurrence of DRM to the antiretroviral drugs implemented in the country at the end of 2004. Of 164 HIV-1 and HIV-2 infected individuals analyzed for drug resistance, 71 and 86 infected patients were ARTn and ARTexp, respectively. For seven patients, this information was not available. The median time on ART for individuals included in this analyses was 23 months (IQR = 1–78) for HIV-1 and 37 months (IQR = 17–60) for HIV-2. Table 3 lists the data for HIV-1 ARTn and HIV-1 and HIV-2 ARTexp. HIV-1 TDRM were observed for 3.4% (CI −1.3% to 8.1%) of the HIV-1 individuals, 1.7% for NRTI and 1.7% for NNRTI. No TDRM was observed in the group of HIV-2 ARTn individuals. Among the 69 HIV-1 patients under ART, 33 (47.8%) presented DRM, 37.7% for NRTI, 37.7% for NNRTI and 7.4% for PI. Twenty-three (33.3%) patients had DRM for two or more drug classes. M184V (69.7%) and K103N (30.3%) were the major NRTI and NNRTI mutations, respectively. A K20I protease mutation, associated with a potential low-level resistance to Nelfinavir, was observed as a typical polymorphism in all subtype G or CRF02_AG sequences obtained from HIV-1-infected patients. One case with DRM to the three drugs classes was observed in an 8 year-old child from Praia infected with subtype G. Of the 33 cases with DRM, 66.7% were women, 36.4% were infected with subtype G, 30.3% with CRF02_AG and 15.6% with subtype B. Among the 17 HIV-2 ARTexp patients, three (17.6%) presented DRMs, all for NRTI and two for PI (Table 3). An M184V mutation was present in all patients with DRMs.

**Table 3:** Prevalence of Drug Resistance Mutations in HIV-1 ART naïve individuals and HIV-1 and HIV-2 patients under ART.

	HIV-1		HIV-2
	ARTn	ARTexp	ARTexp
**Resistance Category N (%)**	58 (45.7)	69 (54.3)	17 (50.0)
**Sequence with any mutation** **N (%)**	2 (3.4)	33 (47.8)	3 (17.6)
Sequence with any PI mutation		5 (7.4)	2 (11.8)
Sequence with any NRTI mutation	1 (1.7)	26 (37.7)	3 (17.6)
Sequence with any NNRTI mutation	1 (1.7)	26 (37.7)	NA
Sequence with two or more drug class mutation		23 (33.3)	2 (11.8)

## Discussion

In this study, we performed a comprehensive molecular characterization of the HIV epidemic in Cape Verde, including samples of HIV-positive individuals from all inhabited islands located in Barlavento and Sotavento regions, except for S. Nicolau and Maio, which together contribute for less than 2.5% of the total HIV cases diagnosed per year in Cape Verde [11]. The HIV epidemic in Cape Verde described in the present study displayed unique characteristics and other features that are similar to those observed in the molecular and epidemiological scenarios for other West African countries.

The two types of viruses circulate in all studied islands in roughly similar proportions. In Praia and “Inner Santiago”, however, both located in the largest and most populated island of Cape Verde (Santiago), the relative frequencies of HIV-1 and HIV-2 infections differed by more than 20%. Although we have no explanation that might clarify this fact, we propose here two hypotheses. First, like in many other countries, the capital and largest Cape Verde city, Praia, was most likely the gateway for the AIDS epidemic, which in the beginning was dominated by HIV-2, accounting for more than 90% of the diagnosed cases in the 80s [13]. Probably, the spread of HIV-2 to other regions of the country occurred later and most likely coincided with the introduction and spreading of HIV-1. Conversely, Inner Santiago, a region with high rates of emigration to Europe, has most likely imported HIV-1 infections by the presence of six of the seven subtype B infections detected in our study group.

Change from predominance of HIV-2 in the beginning of the AIDS epidemics to HIV-1 has been described in West African countries like Senegal, Gambia and Guinea-Bissau [27]–[29], all neighbors of Cape Verde. In Cape Verde, we also observed a relative decrease of HIV-2 compared to HIV-1 over time because the absolute number of HIV-2 cases diagnosed per year remained constant, whereas an increase in the number of HIV-1 cases was observed [11], [13]. Consistent with this trend, we observed that the percentage (44.2%) of HIV-2 infected individuals 49 years or older was much higher than the percentage of HIV-1-infected individuals within that age group (16.6%). In contrast, the proportion of individuals with HIV-1 infections in the age group of 15–29 years old (17.2%) was much higher than the corresponding proportion of HIV-2-infected subjects (7.0%), highlighting the need for prevention programs aimed towards younger people. These data are also consistent with previous studies conducted in other West African countries that observed a higher HIV-2 frequency among individuals over 49 years and a higher HIV-1 prevalence among younger individuals [30]–[32]. Lower frequency of children infected by vertical transmission was observed for HIV-2 (2.3%) compared with HIV-1 (16.0%) infected individuals, as described for other countries where both types circulate [33], [34]. All HIV-2 samples belonged to the predominant group A, as previously described in Cape Verde [18] and other West African countries [35], [36]. Analyzing the HIV-2 group A samples according to the region of origin, we observed the presence of one highly supported cluster that included the majority of samples from the Barlavento region, suggesting possible independent introductions and/or distinct dynamics of the HIV-2 distribution among Cape Verde islands.

The HIV-1 profile described in the present study, covering a large dataset of HIV samples obtained from seven of the 9 inhabited Cape Verde islands, displayed a more complex pattern with a predominance of subtype G (36.6%), followed by CRF02_AG (30.6%) subtype F1 (9.7%), subtype B (5.2%), subtype C (2.2%), and other recombinants forms (15.7%). This subtype distribution is different from that previously estimated in Cape Verde (48% of subtype G, 7% of subtype B, 7% of subtype F1, 7% of CRF02_AG and 30% of other recombinants) [18]. The low number of HIV-1-infected patients analyzed in the previous study (*n* = 27) combined with high complexity of the HIV-1 epidemic in Cape Verde may explain this difference. The HIV-1 molecular epidemiological profile in Cape Verde is also different from that described in other Western African countries where the AIDS epidemic is dominated by CRF02_AG (50%), followed by subtype G (28%), other recombinants (8%) and subtype A (4%) [12]. Thus, the most remarkable molecular characteristics of the Cape Verde HIV-1 epidemic seem to be its high prevalence of subtypes G, CRF02_AG, other recombinant forms, F1 and the absence of the HIV-1 subtype A.

Subtype G, the most prevalent HIV-1 clade in Cape Verde, was observed predominantly in HIV-1 positive patients from the Sotavento region, most likely indicating a local founder effect. In a previous study, Oliveira et al. [18] suggested its origin from Portugal and/or Angola, however, further phylodynamic studies including larger datasets will be necessary to assess the origin of subtype G and its local dispersion. It is important to point out that subtype G is also frequent in some West and Central African countries, including Nigeria, Togo, Cameroon and the Republic of Congo [37]. Other African countries with close relationships with Cape Verde, such as Angola and Guinea-Bissau have also reported the presence of subtype G [38], [39]. Moreover, subtype G is the most prevalent non-B subtype clade in Portugal [40]–[42], a country with close relationships with Cape Verde and a concentrated major emigrant community from Cape Verde in Europe. CRF02_AG, however, seems to be better distributed between the islands, most likely because of independent introductions occurring over time. Although present at an overall low frequency (9.7%), subtype F1 is prevalent in S. Vicente Island, possibly originating from single or few introductions. This subtype was occasionally observed in some West African countries, such as Guinea Conakry, Mali and Senegal [43]–[45], and is frequently detected in other countries with close historical relationships with Cape Verde, such as Angola, Cuba and Brazil [38], [46], [47]. The absence of “pure” HIV-1 subtype A in Cape Verde is notable, although it can be observed in the mosaic genomes observed in some URFs with a AU *pol* mosaic structure. Seven URFs_AU viruses branched in one highly supported monophyletic clade with subtype A, suggesting that they may have evolved from one independent recombinant common ancestor. Three other samples with unrelated AG and AU mosaic structures exhibited sub-subtype A3 in the fragment corresponding to the partial reverse transcriptase region. Sub-subtype A3 is already described in high prevalence in some West African countries, such as Senegal, Guinea-Bissau and Equatorial Guinea [48], [49]. Further studies with whole genome sequences will be necessary to confirm the occurrence of new CRFs in Cape Verde.

Emigration out of Cape Verde has been a reality since the early 20^th^ century. The first Capeverdean emigration wave was to the United States of America. Later, during constant periods of dry and starvation, political instability and conflicts in the 1960s that culminated with its independence in 1975, there was a strong emigration to neighboring West African countries (S. Tomé and Principe, Guinea-Bissau, Senegal and Ivory Coast), South America (Brazil), and Europe (Portugal and Holland). After its independence, emigration from Cape Verde continued predominantly to Europe and the United States, decreasing in the 90s along with the beginning of the economic recession in Europe. In this same time, migration of seasonal workers to Cape Verde from countries such Guinea-Bissau, Senegal, Portugal and Nigeria also occurred [50]. Such displacements to and from Cape Verde have certainly affected the current HIV-1 and HIV-2 genetic diversity in the country, as has been reported in other African countries [51]. Universal access to ART has an important effect on mortality and morbidity, as well as on reducing viral transmission [9]. Conversely, the increase of ART availability provides a challenge on monitoring DRM on ARTn and ARTexp patients and is of great importance in situations where there is intermittent treatment supply or low adherence. In 2004, the ARV became available for all patients with CD4 counts below 200 cells/mm^3^ or those presenting AIDS-defining illness. However, Capeverdean individuals infected with HIV-1 or HIV-2 living in others countries or in Cape Verde may have started their treatment before 2004. Several studies observed increases in TDRM, in particular affecting sub-Saharan Africa, driven by resistance to NNRTI [52], [53]. In the present study, TDRM were observed at low frequency among ARTn HIV-1-infected patients from Cape Verde contrasting with to what has been previously published analyzing a smaller number of drug naïve patients [13]. Our results, however, are in agreement with other studies conducted in resource-limited regions, where ARV programs were scaled up in early- to mid-2000, which have reported that fewer than 5% of new infections are caused by viruses with evidence of transmitted drug resistance [54]–[57]. Moreover, non-significant increase of HIV-1 TDRM over time was described in naïve patients from West Africa [42], [51]. Despite the low prevalence of TDRM in naïve patients from the region, we consider these results of relevance to establish surveillance programs to monitor transmitted DRM in Cape Verde and in the neighbor countries [58].

The prevalence of DRM among Capeverdean ARTexp HIV-1-infected patients (47.8%) enrolled in this study from 2010 to 2011 is higher than the prevalence previously estimated (30%) for this country based on samples collected between 2005 and 2007 [18]. The frequency in Cape Verde is lower than that observed in Senegal [59], but higher than West Africa countries [60] and Portugal [61]. M184V (69.7%) and K103N (30.3%) were the major NRTI and NNRTI mutations, respectively, in Cape Verde, identical to those described in others countries [59], [62].

Although no TDRM was observed among ARTn subjects infected with HIV-2, among the ARTexp, 17.6% presented DRMs, all for NRTI and two for PI. The M184V mutation was present in all ARTexp HIV-2-patients with DRMs. Similar results for DRM were observed in Senegal and Guinea-Bissau [63], [64].

In summary, Cape Verde has a complex HIV molecular diversity epidemic dominated by HIV-1 subtypes G and CRF02_AG and HIV-2 group A. Differences were observed between the subtype distributions among the Cape Verde islands. Recombinant genomes bearing sub-subtype A3 were described for the first time in Cape Verde. Notably, HIV-1 TDRM were observed in the country after only 5–6 years of access to free treatment. The occurrence of TDRM and the relatively high level of DRM among treated patients are of great concern. The continuous monitoring of patients on ARV, including the treatment and the introduction of genotyping, are public policies to be implemented. Moreover, our data also highlight the need to consider non-B subtypes, CRF’s and URF’s a research priority to improve knowledge on pathogenesis, vaccine development and treatment. Studies reporting faster progression to AIDS and death among individuals recently infected with the recombinant virus prevalent in West Africa support this necessity [65]–[68].
